# The impact of job stress on job performance among college interns: the mediating roles of psychological contract and perceived organizational support, and the moderating role of physical and mental health

**DOI:** 10.3389/fpsyg.2026.1914123

**Published:** 2026-08-12

**Authors:** XiaoMing Xie, FengChan Zou, Xinchuang Shi, Tong-Yao Jin

**Affiliations:** 1 Guilin University of Technology at Nanning, Nanning, China; 2 Zhengzhou Tourism College, Zhengzhou, China; 3 Suan Sunandha Rajabhat University, Bangkok, Thailand

**Keywords:** conservation of resources theory, job performance, job stress, moderated mediation, perceived organizational support, physical and mental health, psychological contract

## Abstract

**Objective:**

College student interns frequently experience transitional stress as they navigate the shift from campus life to internship environments. Coping strategies for this transition have garnered attention within the field of organizational behavior. However, the current literature remains insufficient in examining the contingency mechanisms of internal psychological resources and external organizational support. Grounded in the Conservation of Resources Theory, this study aims to construct and test a moderated parallel dual-mediation model to elucidate how Physical and mental health (PMH) be associated with the divergent pathways linking job stress (JS) to job performance (JP).

**Methods:**

This study employed partial least squares structural equation modeling (PLS-SEM) and bootstrapping (with 5,000 resampling iterations) to perform empirical tests and simple slope analyses on data collected from 464 interns in Guangxi Province, China.

**Results:**

The results indicate that the direct effect of JS on JP was not significant; however, a significant parallel mediating effect was observed through psychological contract (PC) and perceived organizational support (POS). A core finding of this study is that PMH was associated with a divergent moderation pattern across the dual mediation paths. Specifically, when individuals’ PMH levels were low, the PC pathway was significantly stronger; conversely, when PMH levels were high, the POS pathway became dominant, effectively translating external resources into performance. In conclusion, this study extends existing stress-performance frameworks by demonstrating that the mechanism through which JS be associated with performance is contingent on individuals’ PMH, offering new insight into interns’ resource allocation preferences under varying health conditions.

**Conclusion:**

This research expands the application boundaries of Conservation of Resources theory within the realm of occupational health and provides an empirical foundation for organizations to adopt “health-first” and “differentiated intervention” human resource management strategies.

## Introduction

In recent years, the ongoing expansion of higher education in China has intensified competition in the graduate labor market. As the system transitions from an elite to a more universal model of higher education, the disparity between the supply and demand for graduates has become increasingly pronounced (Long, 2025; Ye, 2025). Initial employment rates have declined from approximately 90% at the turn of the century to between 70 and 80% today (Xiang et al., 2023), reflecting a complex and uncertain employment landscape for contemporary youth entering the labor market.

In this context, an increasing number of universities have recognized internships as a critical pathway for enhancing students’ employability and facilitating their transition into the workforce. Serving as a bridge between higher education and employment, internships enable students to translate classroom knowledge into practical competencies while fostering professional skills, industry awareness, and workplace adaptability (Bawica, 2021). High-quality internship experiences have been shown to significantly improve graduates’ employability and career competitiveness (Pianda et al., 2024; Thakur et al., 2024). However, this transition is often fraught with challenges: university interns frequently encounter substantial stress arising from unfamiliar tasks, heavy workloads, limited autonomy, inadequate remuneration, and the multiple demands imposed by supervisors (Joseph et al., 2022; Jackson and Li, 2022). This imbalance between the resources available to interns and the demands they face tends to particularly burden less experienced individuals, who require additional time to adapt to unfamiliar organizational settings (Ibrahim et al., 2025; Ikeda, 2023).

Despite the growing recognition of these challenges, the association between job stress (JS) and the workplace behavior and performance of university interns during this critical career transition remains insufficiently understood (Azila-Gbettor et al., 2022). A more comprehensive understanding of the mechanisms through which stress affects this population carries substantial practical importance. To address this gap, the following section reviews relevant theoretical perspectives and develops the hypothesized relationships that underpin this study.

## Literature review and hypotheses

### Job stress and job performance

In organizational behavior, job performance (JP) is regarded as a fundamental variable for assessing employees’ contributions to organizational objectives (Ramos-Villagrasa et al., 2019; Marcus and Schuler, 2004; Bommer et al., 1995). Numerous empirical studies have demonstrated that excessive JS can trigger a series of adverse effects, including emotional distress, physical and mental exhaustion, and various health issues, which subsequently lead to increased organizational costs and reduced efficiency (Schulte et al., 2024; Hassard et al., 2018; Beehr and Newman, 1978). When individuals encounter excessive JS, it not only undermines their physical and mental well-being but can also directly impair their JP (Nugroho, 2023; Chen et al., 2022; Parisuda and Dewi, 2021; Ganster and Rosen, 2013). Employees subjected to high pressure may experience diminished performance due to psychosomatic disorders.

Excessive stress can lead to information overload, causing employees to redirect their attention and mental energy away from essential tasks, thereby reducing their performance capabilities (Parra-Medina and Alvarez-Cervera, 2021; Jackson and Farzaneh, 2012). Some studies have reported a correlation coefficient as high between stress and performance decline, indicating that stress is a significant predictor of performance (Meunier et al., 2022; Gilboa et al., 2008). Under high stress, employees often struggle to concentrate, which can result in negative emotions such as workplace dissatisfaction (Cotos-Gamarra et al., 2023). However, the relationship between stress and performance is not merely linear; numerous studies have shown that JS can positively correlate with JP under certain conditions (Djauhar et al., 2022; Hashemnia et al., 2014). Stress can act as both a motivator and a guiding force for motivation (Peranginangin et al., 2024); moderate levels of stress can unlock employee potential and enhance motivation. This divergence in research findings suggests that the relationship between stress and performance is highly complex, necessitating consideration of other moderating or mediating variables.

The complexity of the stress-performance relationship has been addressed by several established frameworks. The challenge-hindrance stressor framework (Cavanaugh et al., 2000) distinguishes job stressors based on their potential to facilitate growth and achievement or impede goal attainment. The Job Demands-Control (JDC) model (Karasek Jr, 1979) further suggests that job control can mitigate the adverse effects of high job demands. Extending this perspective, the Job Demands-Resources (JD-R) model (Demerouti et al., 2001; Bakker and Demerouti, 2007) posits that both job resources and personal resources alleviate the strain associated with excessive job demands while promoting motivation and performance. Although these frameworks have significantly enhanced our understanding of when and how stress relates to performance, they have primarily been developed and validated in the context of established employees, offering comparatively limited insights into interns, who face distinct challenges during their transition from academic environments to professional workplaces. Specifically, there is a lack of understanding regarding how stress influences interns’ work performance through their unique perceptions, limited organizational experience, and evolving comprehension of the employer-employee relationship.

The present study extends this line of inquiry by focusing on psychological contract (PC) and perceived organizational support (POS) as the primary mediating mechanisms. This approach highlights a relational-cognitive pathway that complements the resource- and control-based mechanisms emphasized in previous frameworks. However, considering that university interns typically have limited coping resources, professional experience, and organizational tenure compared to established employees, the hindrance-oriented aspects of JS are likely to outweigh its potential motivational effects within this population. Therefore, we anticipate that JS will be associated with an overall negative impact on JP among university interns.

### The mediating role of psychological contract and perceived organizational support

According to social exchange theory, an individual’s cognitive assessment of “relational reciprocity” within an organization serves as a crucial mechanism explaining how JS translates into behavioral outcomes. Within this framework, PC and POS represent two fundamental cognitive pathways through which JS may shape JP.

PC underscore the unwritten agreements and mutual commitments between employers and employees (Liu and Liu, 2020; Cheng, 2021; Mensah et al., 2021; Maia and Bastos, 2019). When intense workplace stress disrupts the reciprocal balance perceived by new employees, feelings of PC breach are likely to emerge, as employees may perceive that the organization has failed to honor its implicit obligations under conditions of heightened demand. Conversely, when the PC is well-fulfilled, employees tend to demonstrate higher work motivation, which in turn leads to stronger JP. In contrast, breaches or non-fulfillment of this contract are associated with increased JS, dissatisfaction, and diminished performance (Zhang et al., 2025; Yu et al., 2022). Collectively, these arguments suggest that JS may undermine employees’ perceptions of psychological contract fulfillment, subsequently contributing to reduced JP.

Similarly, POS—defined as employees’ overall perception of how much the organization values their contributions and cares for their well-being (Bilginoğlu and Yozgat, 2018; Eisenberger et al., 1986)—represents a second cognitive pathway linking JS to JP. Under conditions of high JS, employees are more likely to interpret the organization’s response, or lack thereof, as a signal of insufficient care, thereby diminishing their POS. Conversely, high levels of POS indicate to employees that they can expect emotional support and assistance from the organization when faced with significant work demands (Bonaiuto et al., 2022; Jung and Yoon, 2013; Loi et al., 2014), which helps mitigate the negative perceptions associated with a lack of care or assistance (van Zoonen et al., 2021; Ha and Kim, 2021) and, in turn, sustains or enhances employees’ JP (Cai et al., 2024; Fajri et al., 2022). Accordingly, JS may also negatively affect POS, which is subsequently associated with a positive correlation with JP.

These mechanisms may be particularly salient among university interns. Unlike established employees, interns typically possess limited organizational experience, weaker occupational identity, and greater uncertainty regarding organizational expectations (Peng et al., 2024; Rose, 2018). Consequently, their evaluations of reciprocity and organizational support may be more sensitive to stressful workplace experiences, making PC and POS critical mechanisms linking JS to performance outcomes.

While social exchange theory elucidates employees’ cognitive evaluations of relational reciprocity, these evaluations also reflect their perceptions of available relational and emotional resources. From the perspective of Conservation of Resources theory, fulfilled psychological contracts and strong organizational support represent valuable relational resources, whereas breaches of contracts and insufficient support signify resource loss, which further diminishes employees’ capacity to cope with workplace demands. This resource-based perspective serves as a theoretical bridge to the subsequent discussion of physical and mental health (PMH) as a boundary condition that shapes these processes.

### Conservation of resources theory and the moderating role of physical and mental health

Building on the resource-based perspective introduced above, individual PMH can be understood as the cornerstone of employees’ internal resource pool, underpinning their overall resilience to external stress. The World Health Organization emphasizes that PMH is crucial for an individual’s ability to cope with stress and work effectively; healthy employees demonstrate higher motivation, focus, job satisfaction, and enhanced JP (Chang, 2024; Peláez Zuberbühler et al., 2023; Lohmann et al., 2019). Empirical research indicates that employees with superior PMH exhibit greater resilience to stress-related performance declines (Jia et al., 2022). Conversely, negative and stressful work environments can precipitate mental health disorders, such as psychological distress, anxiety, and depression, which significantly contribute to productivity loss and illness-related absenteeism, thereby exerting a cascading negative impact on performance in both work and social contexts (Nwobodo et al., 2023; Meunier et al., 2022; Gellert et al., 2021; Agyemang et al., 2014). Only workers who do not endure prolonged excessive stress can sustain their PMH while delivering optimal JP (Vulanović et al., 2020), and the pathways through which psychological and physical stress affect performance outcomes are uniquely distinct (Siswadi et al., 2024).

Psychological Capital (PMH) reflects the overall level of an individual’s internal resource reserves. Employees with higher PMH are better equipped to withstand the resource depletion associated with a breached PC or diminished POS. In other words, when core internal resources are abundant, the negative implications of a compromised PC or reduced POS are less likely to translate into a decline in performance. Conversely, employees with depleted PMH may lack the necessary reserves to buffer against these relational resource losses. Therefore, PMH plays an indispensable role in regulating and safeguarding the strength of the pathways linking JS, its cognitive mediators, and performance outcomes. Collectively, these arguments suggest that PMH serves as a boundary condition that shapes the strength of the indirect effects of JS on JP through PC and POS.

### The present study

While academia has demonstrated considerable interest in workplace transitions and stress management, few studies have integrated external organizational resources (POS), internal contractual beliefs (PC), and individual *resource conditions* (PMH) into a unified model specifically designed to examine university interns. Previous research has primarily concentrated on traditional high-pressure industries, such as healthcare, or on established employees (Morsy and Ebraheem, 2020; Tehreem et al., 2023), leading to a limited understanding of the psychological dynamics and behavioral responses of interns under stress. This study addresses this gap by extending the dual-mediation framework to a population characterized by limited organizational tenure and coping resources. It simultaneously integrates external organizational support, internal relational cognition, and individual health resources within a single moderated mediation model.

**Figure 1:**
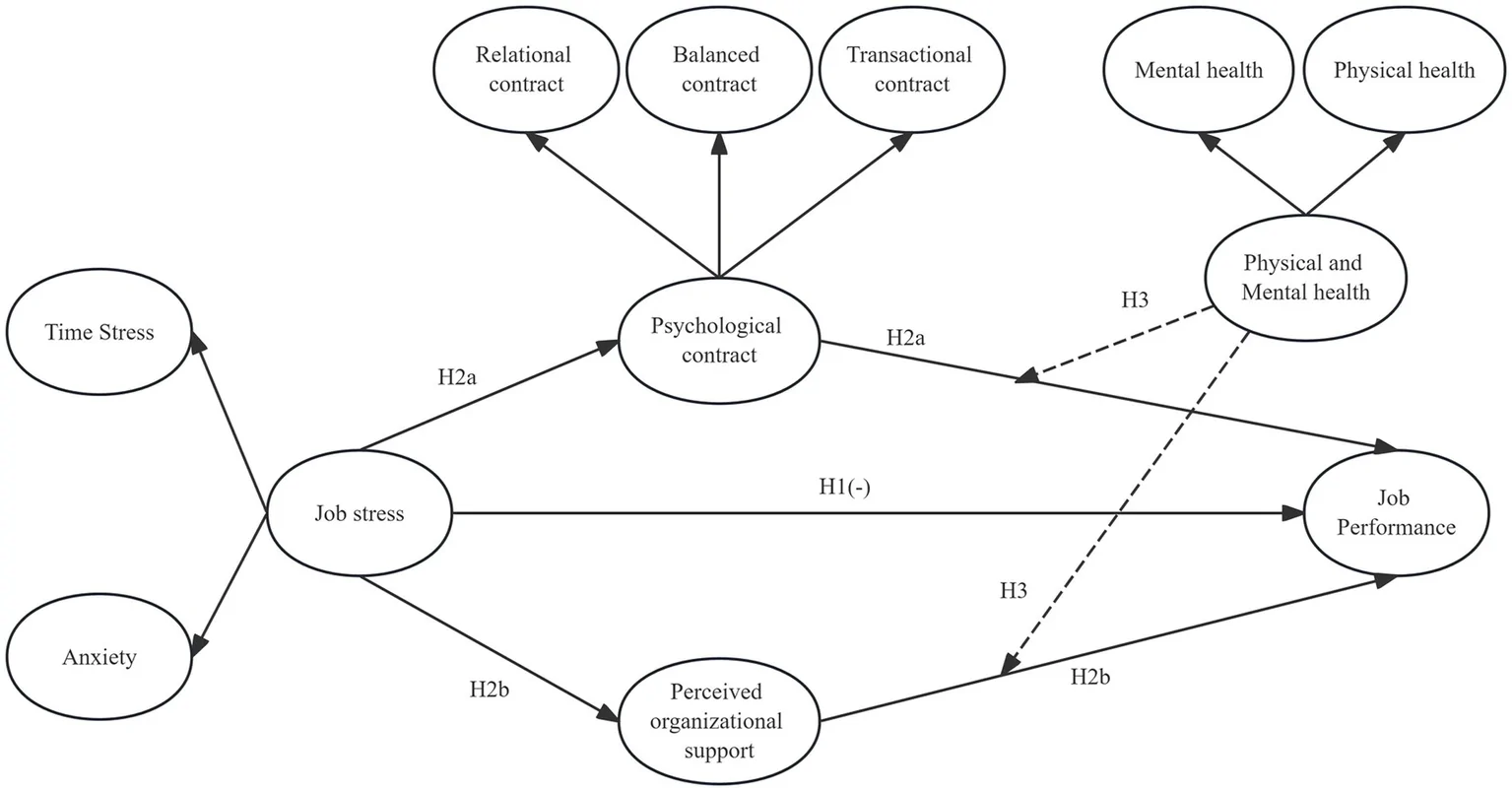
Research model.

This study, grounded in social exchange theory and resource conservation theory, aims to explore the intrinsic mechanisms through which JS be associated with the JP of university interns. It specifically focuses on verifying the dual mediating effects of the PC and POS, as well as the conditional moderating role of PMH, as illustrated in Figure 1. This study proposes the following core hypotheses:

*H*1: Job stress negatively predicts the job performance of university interns.

*H*2a: Job stress negatively predicts psychological contract fulfillment, which in turn positively predicts job performance, indicating a mediating role of psychological contract.

*H*2b: Job stress negatively predicts perceived organizational support, which in turn positively predicts job performance, indicating a mediating role of perceived organizational support.

*H*3: Physical and mental health moderates the indirect effects of job stress on job performance via psychological contract and perceived organizational support.

## Method

### Participants

This study employed a purposive sampling approach to collect 473 samples from higher education institutions in Nanning, Guangxi Province. After excluding 9 samples from individuals under 18 years of age, a final sample of 464 participants from various academic levels and majors was obtained. Participants were recruited throughout the data collection period, and all students who met the inclusion criteria and consented to participate were incorporated into the final sample. Eligibility for participation included: (a) being a formally enrolled higher education student (intern) during the sampling period; and (b) the ability to complete the questionnaire without cognitive or language comprehension impairments. Appropriate exclusion criteria were implemented to ensure data quality: (a) individuals under 18 years of age; (b) participants who did not complete their demographic information; and (c) participants exhibiting obvious response patterns or unusual response times. These criteria were assessed and filtered through a post-collection data cleaning process. Furthermore, participants had the right to withdraw from the study at any time without facing any consequences.

In terms of demographic characteristics, the final sample (*N* = 464) comprised 225 males (48.5%) and 239 females (51.5%), indicating a relatively balanced gender distribution. Regarding educational background, 259 students (55.8%) were enrolled in associate degree programs, while 205 students (44.2%) pursued bachelor’s degrees. Concerning academic major, 153 students (33.0%) were in the humanities, whereas 331 students (67.0%) were in the sciences. This research protocol has been formally reviewed and approved by the Ethics Committee of Guilin University of Technology, Nanning Branch (No. GLLGD20241230031). All participants were fully informed of the research objectives prior to their participation and provided electronic informed consent by completing a consent selection before proceeding to the questionnaire.

### Psychometric instruments

#### Job stress scale

The Job stress scale was developed by Jamal and Baba (1992) and initially demonstrated a total scale reliability of 0.83 (Cronbach’s *α*). Following the revision approach of Shukla and Srivastava (2016), certain items were adaptively adjusted. To enhance the scale’s applicability to our sample, statements from the original scale that were specific to certain occupational backgrounds were modified to better suit general workplace situations. The revised scale consists of nine items, divided into two dimensions: Time Stress (4 items) and Anxiety (5 items). A 5-point Likert scale was utilized, ranging from 1 (strongly disagree) to 5 (strongly agree), with higher scores indicating a greater perceived level of JS. A representative item is: “I have a lot of work, but I am concerned that I will not have enough time to complete it.”

#### Job performance

The Self-Assessment of Job performance scale was employed to evaluate job performance, utilizing a simplified version of the Self-Assessment of Job performance scale (SJoP) developed by de Azevedo Andrade et al. (2020). This scale is a modification of the original 20-item scale proposed by Queiroga (2009), which has been condensed to 10 items. Although the theoretical framework of the scale was designed to capture two dimensions—task performance (5 items) and situational performance (5 items)—de Azevedo Andrade et al. (2020) reported a significant overlap between these dimensions (r = 0.83) and very low reliability of the factors after controlling for general factors. In accordance with the authors’ recommendation, this study treated the scale as a single-dimensional, reflective construct. All items were rated on a five-point Likert scale, ranging from 1 (never) to 5 (always), with higher scores indicating a greater perceived JP. Representative items included “I am able to effectively complete difficult tasks” and “I take proactive steps to improve my JP.”

#### Psychological contract

Psychological contract (PC) was measured using the Chinese version of the Psychological contract Inventory validated by Hui et al. (2004), which captures the transactional, relational, and balanced dimensions of Psychological contracts within the Chinese organizational context. This study adhered to the research framework established by Hui et al. (2004), utilizing three theoretical dimensions—relational contract, balanced contract, and transactional contract—to assess the PC. Given the extensive number of items in the original scale and the necessity of ensuring its relevance to the sample, an exploratory factor analysis (EFA) was initially performed to investigate the potential factor structure of the scale within the current dataset and evaluate item fit. The results indicated that several items exhibited significant cross-factor loadings or factor loadings that fell below the minimum threshold of 0.50. To enhance the construct validity of the scale and achieve a coherent factor structure, the research team removed these non-compliant items in accordance with established psychometric standards. Ultimately, the retained items resulted in a distinct three-factor structure, with all items demonstrating significant loadings on their respective factors and minimal cross-loadings.

#### Perceived organizational support

The Perceived organizational support scale was evaluated using the classical scale developed by Wayne et al. (1997), which consists of nine reflective items. This scale utilizes a 5-point Likert scale, ranging from 1 (strongly disagree) to 5 (strongly agree), where higher scores indicate a stronger perception of organizational support. Representative items include, “The organization genuinely cares about my well-being” and “The organization significantly considers my goals and values.” In the original study, the scale demonstrated a Cronbach’s *α* coefficient of 0.919, indicating excellent internal consistency reliability.

#### Physical and mental health

This study utilized a simplified version of the Occupational Stress Index-2 (OSI-2), developed by Lu and Gao (1999), which focuses on two dimensions: ‘Mental Health’ and ‘Physical Health’. These dimensions were employed to assess the mental and physical well-being of university interns. The scale consists of 10 items, evaluated using a 5-point Likert scale, with responses ranging from 1 (strongly disagree) to 5 (strongly agree). A higher overall score indicates poorer PMH conditions, reflecting a greater degree of mental and physical distress. Previous research has established that this scale exhibits strong reliability, as evidenced by an overall Cronbach’s α coefficient of 0.88. The internal consistency reliability for the ‘Mental Health’ dimension ranged from 0.83 to 0.86, while the ‘Physical Health’ dimension ranged from 0.82 to 0.89 (Gao and Lu, 2011). This indicates that the physical and mental health scale employed in this study demonstrates robust measurement reliability.

#### Procedure

Before officially commencing data collection, the research team rigorously adhered to the relevant communication and ethical review procedures. Initially, researchers contacted the student affairs departments and internship management personnel of the pertinent universities via email and telephone, detailing the core objectives of the research and providing a comprehensive explanation of data confidentiality agreements and anonymization measures. After obtaining departmental permission and reaching a consensus, the research team organized a unified schedule for data collection.

Data collection for this study was conducted intensively in January 2025. Prior to the official distribution of questionnaires, researchers provided prospective interns with a comprehensive overview of the research background and specific objectives. To ensure that participants were fully aware of their legal rights, all participants provided informed consent by ticking a consent checkbox at the beginning of the online questionnaire, after being informed of the voluntary nature of the research and their right to withdraw at any time (Only those who indicated consent were able to proceed to the survey items). After completing the informed consent process, each participant independently filled out a comprehensive questionnaire that encompassed demographic characteristics and core measurement tools, including the job stress scale, Psychological contract scale, Perceived organizational support scale, Job performance scale, and Physical and mental health scale.

To minimize the potential correlate with of social desirability bias on the measurement results, researchers clearly articulated the purely academic nature of the research in the questionnaire instructions, emphasizing that the study aimed to objectively explore the genuine experiences and psychological states of university students during their internships. Additionally, the researchers reiterated to participants that there were no standard or correct answers for any of the scale items and that all responses would be kept strictly confidential and used solely for overall statistical analysis, thereby minimizing participants’ psychological concerns and ensuring the authenticity and validity of the data. Thereby minimizing participants’ psychological concerns and ensuring the authenticity and validity of the data.

Before conducting formal statistical analysis, the average scores of the corresponding scale items were calculated to derive comprehensive scores for JS, PC, POS, JP, and PMH. Higher scores reflect a deeper understanding of these constructs. Data analysis in this study was performed using SPSS 26.0 and Smart PLS 4 software. To mitigate potential interference, participants’ demographic characteristics were included as control variables in the initial analysis phase. Subsequently, a structural model was developed using Smart PLS 4 to examine the direct be associated with of JS (independent variable) on JP (dependent variable), along with the dual mediating effects of perceived PC and POS. During this stage, the bootstrapping method (with 5,000 resamples) was employed to calculate the indirect effects and their 95% confidence intervals, thereby assessing the significance of the mediating effects. In the third step, to evaluate the moderating effect of PMH, the main effects of PMH, along with their two-way interaction terms with relevant antecedent variables in the model, were incorporated into the structural model. The change in the model’s explanatory power (R^2^) following the introduction of the interaction term, as well as the significance of the interaction term’s path coefficient, were utilized to reflect the moderating contribution of PMH to the model. For significant interaction effects, the study further conducted simple slope analysis under conditions of low (−1 standard deviation) and high (+1 standard deviation) PMH to elucidate the specific direction and strength of the moderating effect across various scenarios.

## Results

This study employed partial least squares structural equation modeling (PLS - SEM) as the primary statistical analysis technique. Given that our model incorporates multiple latent variables, dual mediation, and moderating pathways, PLS - SEM is particularly suitable for exploring and empirically validating complex theoretical models due to its relatively lenient requirements regarding the normality of the sample distribution (Hair et al., 2021; Henseler et al., 2016). In the measurement model evaluation phase, Smart PLS4 was utilized to assess internal consistency reliability, convergent validity, and discriminant validity for each core construct, thereby confirming the reliability and effectiveness of the measurement tool. During the structural modeling and hypothesis testing phase, path coefficients were accurately estimated using Smart PLS4 and its Bootstrap self-sampling method (with 5,000 repeated samplings). This phase not only verified the dual mediating effect of perceived PC and POS but also examined the moderating effect of PMH within the model, supplemented by simple slope analysis, thus rigorously validating the theoretical hypotheses proposed in this study.

### Descriptive statistics

Table 1 presents the descriptive statistics, including the mean and standard deviation, for all core constructs in this study. Each construct was measured using a five-point Likert scale, with a theoretical median of 3. Consequently, the mean value directly reflects the average perception or performance level of the respondents regarding specific variables.

**Table 1:** Descriptive statistics of key constructs (N = 464).

Variable name	M	SD
Job stress (JS)	2.500	0.761
Job Performance (JP)	3.664	0.751
Perceived Organizational Support (POS)	3.300	0.745
Psychological Contract (PC)	3.088	0.593
Physical and Mental Health (PMH)	2.755	0.841

All surfaces were rated on a five-point Likert scale (1 = strongly disagree, 5 = strongly agree). Higher scores indicate a higher level of perception or expression of that surface.

The data results indicate that respondents achieved the highest scores in JP (M = 3.664, SD = 0.751). In contrast, the mean scores for POS (M = 3.300, SD = 0.745) and PC (M = 3.088, SD = 0.593) were both above the theoretical median of the scale. This suggests that, overall, this group of university student interns exhibited commendable JP and maintained a moderately positive perception of the support offered by the organization, as well as the PC between the parties involved.

The mean score for PMH was 2.755 (SD = 0.841), while the mean score for JS was the lowest at 2.500 (SD = 0.761), both of which fall below the theoretical median of 3.0. This finding indicates that respondents perceived their overall JS to be at a moderately low level. However, it is important to note that their self-assessments of PMH were also relatively low, demonstrating a somewhat weak trend. The standard deviations of all core constructs ranged from 0.593 to 0.841, suggesting that the dispersion of data within the sample was moderate. This indicates that the respondents’ subjective evaluations were relatively concentrated, with minimal extreme variation, thereby providing a solid foundation for subsequent structural equation modeling (PLS-SEM) path analysis.

### Two-variable correlation analysis

Table 2 presents the Pearson correlation coefficient matrix among the core variables in this study. Overall, the correlation directions among the variables align with theoretical expectations, providing preliminary empirical evidence for the subsequent structural equation modeling (PLS-SEM) testing.

**Table 2:** Correlation.

Variable name	JS	JP	POS	PC	PMH
Job stress (JS)	1				
Job Performance (JP)	−0.170**	1			
Perceived Organizational Support (POS)	−0.310**	0.393**	1		
Psychological Contract (PC)	−0.243**	0.390**	0.713**	1	
Physical and Mental Health (PMH)	0.660**	−0.160**	−0.339**	−0.256**	1

**At the 0.01 level (two-tailed), the correlation is significant.

The results of the association analysis between the independent variable and the dependent variable, as well as the mediating variables, indicate that the independent variable, JS, is significantly negatively correlated with the dependent variable, JP (*r* = −0.170, *p* < 0.01), thereby providing preliminary support for the hypothesized negative predictive effect of JS on JP. Concurrently, JS exhibits significant negative correlations with both mediating variables—POS and PC (*r* = −0.310, *p* < 0.01 and *r* = −0.243, *p* < 0.01, respectively).

Regarding the association between the mediating variables and the dependent variable, both POS and PC demonstrated significant positive correlations with JP, with correlation coefficients of *r* = 0.393 (*p* < 0.01) and *r* = 0.390 (*p* < 0.01), respectively. These findings align with theoretical expectations, indicating that higher levels of POS and stable PC may enhance JP among interns.

In terms of moderating variables, PMH exhibited significant associations with all other variables in the model. Specifically, PMH showed a strong positive correlation with JS (*r* = 0.660, *p* < 0.01), while it demonstrated significant negative correlations with JP (*r* = −0.160, *p* < 0.01), POS (*r* = −0.339, *p* < 0.01), and PC (*r* = −0.256, *p* < 0.01). This correlation network underscores the statistical validity of incorporating PMH as an intervention factor within this model.

The correlation coefficient between POS and PC was notably high in this study (*r* = 0.713, *p* < 0.01). This significant correlation indicates a robust synergistic relationship between the two constructs within organizational behavior concepts; however, it also raises concerns regarding potential multicollinearity in subsequent multivariate analyses. While the bivariate correlation analysis preliminarily validated the associations between these constructs, the Pearson correlation coefficient does not account for the cross-influence of other variables. Therefore, in the subsequent phase of evaluating the PLS-SEM structural model, this study will rigorously assess multicollinearity using the variance inflation factor (VIF) and will conduct more robust path estimation and hypothesis testing for complex direct effects, dual mediation effects, and moderating mechanisms.

### Reliability

Internal consistency reliability was assessed primarily using Cronbach’s *α*, rho_a, and CR. According to Hair et al. (2019), the ideal critical values for these reliability indicators should exceed 0.70. The PC was conceptualized as a higher-order reflective construct that encompasses three first-order dimensions: relational, balanced, and transactional contracts. This framework aligns with established conceptualizations of the structure of psychological contracts. In accordance with standard practices in Partial Least Squares Structural Equation Modeling (PLS-SEM), the estimation of the higher-order construct was conducted using the disjoint two-stage approach, as outlined by Hair et al. (2019).

The data indicated that, with the exception of the PC construct, the Cronbach’s *α* values for the other constructs ranged from 0.845 to 0.932, which is considered excellent. Regarding the Cronbach’s α value for the PC construct (0.592), which fell below the conventional threshold of 0.70, this is likely attributable to the higher-order nature of the construct: because Cronbach’s α assumes a single underlying dimension and equal indicator loadings, it is known to systematically underestimate reliability when applied to composite indicators derived from multidimensional constructs (Hair et al., 2019). Consistent with this explanation, the rho_a value (0.815) and CR value (0.786) for PC—both of which do not rely on the tau-equivalence assumption and are therefore more appropriate for evaluating higher-order constructs in PLS-SEM—exceeded the 0.70 threshold, as did its AVE (0.593), which exceeded the 0.50 threshold. Across all constructs, rho_a values ranged from 0.815 to 0.936 and CR values ranged from 0.786 to 0.947, all exceeding the acceptance criterion of 0.70, indicating adequate internal consistency across the measurement model (Table 3).

**Table 3:** Reliability and convergent validity assessment results of the measurement model.

Variable name	Cronbach α	rho_a	CR	AVE
Job stress (JS)	0.845	0.845	0.928	0.865
Job performance (JP)	0.932	0.936	0.942	0.621
Perceived organizational support (POS)	0.905	0.912	0.925	0.637
Psychological contract (PC)	0.592	0.815	0.786	0.593
Physical and Mental health (PMH)	0.887	0.887	0.947	0.899

Convergent validity was evaluated using Average Variance Extracted (AVE), where values of 0.50 or higher signify that a latent variable explains more than half of the variance in its indicators. As illustrated in Table 4, the AVE values for all constructs ranged from 0.593 (PC) to 0.899 (PMH), surpassing the 0.50 threshold and thereby confirming adequate convergent validity throughout the measurement model.

**Table 4:** Discriminant validity assessment results of the measurement model.

Variable name	JP	JS	PC	PMH	POS
Heterotrait–monotrait ratio					
Job stress (JS)					
Job Performance (JP)	0.160				
Perceived Organizational Support (POS)	0.467	0.335			
Psychological Contract (PC)	0.156	0.761	0.407		
Physical and Mental Health (PMH)	0.395	0.361	0.887	0.390	
Fornell–Larcker criterion					
Job stress (JS)	**0.788**				
Job Performance (JP)	−0.145	**0.930**			
Perceived Organizational Support (POS)	0.393	−0.244	**0.770**		
Psychological Contract (PC)	−0.143	0.659	−0.271	**0.948**	
Physical and Mental Health (PMH)	0.383	−0.321	0.729	−0.352	**0.798**

In the Fornell-Larcker criterion, the bolded diagonal values are the square root of the average sample variance (AVE) for each construct, and the off-diagonal values are the correlation coefficients between constructs.

In summary, although the Cronbach’s α for the latent variable PC fell below the conventional threshold—a pattern that aligns with its classification as a higher-order construct—the corresponding rho_a, CR, and AVE values demonstrate that its reliability and convergent validity are acceptable within the PLS-SEM framework. Consequently, the measurement model as a whole satisfies the essential prerequisites for the subsequent evaluation of discriminant validity and the testing of the structural model.

### Discriminant validity

To ensure sufficient empirical discriminative power among the latent variables in this study, we employed a dual assessment of discriminative validity using both the traditional Fornell-Larcker criterion and the more recent Heterotrait-Monotrait Ratio (HTMT; Table 4).

According to the Fornell-Larcker criterion, the square root of the average variance extracted (AVE) of a latent variable should exceed the correlation coefficients between that latent variable and all other latent variables in the model. As illustrated in the lower half of Table 5, the bolded values on the diagonal of the matrix (i.e., the square roots of the AVE for each construct, ranging from 0.770 to 0.948) consistently exceed the off-diagonal values in their respective rows and columns (i.e., the Pearson correlation coefficients between constructs). This observation preliminarily indicates that the constructs exhibit statistically acceptable independence.

**Table 5:** VIF values for collinearity diagnostics of measurement indicators.

JS	VIF	PC	VIF	POS	VIF	JP	VIF	PHM	VIF
TS1	1.576	RL3	2.756	POS2	1.576	JP1	1.875	MH1	1.935
TS2	1.680	RL4	1.565	POS3	2.782	JP2	2.343	MH2	2.313
TS3	1.139	RL5	2.132	POS4	3.072	JP3	2.150	MH3	2.323
TS4	1.266	RL6	3.083	POS5	3.216	JP4	2.754	MH4	2.089
AS1	2.189	RL7	3.350	POS7	2.214	JP5	2.766	MH5	2.662
AS2	2.006	RL8	3.293	POS8	2.379	JP6	2.332	MH6	2.383
AS3	2.754	TC1	1.201	POS9	1.959	JP7	2.659	PH1	1.948
AS4	2.587	TC2	1.160			JP8	2.397	PH2	2.040
AS5	1.551	TC3	1.369			JP9	2.375	PH3	1.255
		TC4	1.383			JP10	2.311	PH4	1.512
		TC5	1.461						
		TC6	1.324						
		BL1	2.271						
		BL2	2.687	BL7	3.329				
		BL3	1.350	BL8	2.968				
		BL4	2.887	BL9	3.241				
		BL5	3.146	BL10	2.666				
		BL6	2.908	BL11	3.159				

Recent methodological studies have indicated that the Fornell-Larcker criterion may lack sufficient sensitivity in detecting discriminant validity. To address this limitation, this study introduces the HTMT ratio, as recommended by Henseler et al. (2015), as a more stringent criterion. Generally, for constructs with some conceptual affinity, the acceptable threshold for the HTMT value is set at 0.90. The data presented in the upper part of Table 5 indicate that the HTMT values between the vast majority of latent variables are well below the stringent standard of 0.85. Notably, the HTMT value between PC and POS is 0.887. While this value is relatively high, reflecting the conceptual closeness between the two within the framework of organizational behavior theory, it remains below the upper limit of the highest threshold of 0.90.

In summary, whether evaluated using the Fornell-Larcker criterion or the more stringent HTMT ratio, the measurement model in this study demonstrates satisfactory discriminant validity.

### Common method bias

Harman’s single-factor test was conducted to evaluate the potential presence of common method bias. All items from the five constructs were included in an unrotated principal component factor analysis. The results indicated the presence of seven factors with eigenvalues greater than 1, with the first (unrotated) factor accounting for 28.391% of the total variance, which is significantly below the recommended threshold of 50% (Podsakoff et al., 2012). These findings suggest that common method bias is unlikely to pose a substantial threat to the validity of the results in this study.

### Collinearity test

To avoid distorting the accuracy of path coefficient estimation due to multicollinearity, this study rigorously examined the VIF for all predictor variables in the model, in accordance with the recommendations of Hair et al. (2019). It is widely accepted in academia that excessively high VIF values (greater than 5 or 10) indicate a significant degree of linear correlation between variables, which can compromise the stability of the model structure. The data presented in Table 5 clearly demonstrate that the VIF values for all indicators in this model are significantly lower than the safe threshold of 5. Therefore, we conclude that the data in this study do not exhibit extreme multicollinearity bias, and the independence between variables remains within an acceptable range, ensuring that it will not hinder subsequent empirical analysis and hypothesis derivation.

### Explanatory power and predictive relevance

Following the establishment of the reliability and validity of the measurement model, this study further evaluated the explanatory power and predictive relevance of the structural model. As shown in Table 6, model quality was assessed using the coefficient of determination (R^2^), the adjusted R^2^, and predictive relevance (Q^2^). R^2^ reflects the proportion of variance in an endogenous construct explained by its predictors; following Hair et al. (2011), values of 0.75, 0.50, and 0.25 correspond to strong, moderate, and weak explanatory power, respectively.

**Table 6:** Explanatory power (R^2^) and predictive relevance (Q^2^) of the structural model.

Path relationship	Coefficient of determination(R^2^)	R^2^ adjusted	Predictive relevance(Q^2^)
Job stress (JS)	-	-	-
Job Performance (JP)	0.210***	0.199***	0.006
Perceived Organizational Support (POS)	0.058***	0.056***	0.052
Psychological Contract (PC)	0.103***	0.101***	0.096

***At the 0.01 level (two-tailed), the correlation is significant.

As shown in Table 6, the R^2^ value for JP was 0.210 (*p* < 0.001), approaching the conventional threshold of 0.25, indicating a modest but statistically significant level of explained variance (21.0%). The R^2^ value for PC was 0.103 (*p* < 0.001), and the R^2^ value for POS was 0.058 (p < 0.001). Although the explanatory power for PC and POS was relatively modest, such levels retain theoretical and exploratory value within social science and organizational behavior research. The corresponding adjusted R^2^ values (JP = 0.199; PC = 0.101; POS = 0.056) were close to their unadjusted counterparts, supporting the robustness of the model and suggesting a low risk of overfitting.

To assess the out-of-sample predictive quality of the model, a blindfolding procedure was used to compute Q^2^ for each endogenous construct. A Q^2^ value greater than 0 indicates that the structural model has predictive relevance for that construct (Hair et al., 2014). As shown in Table 6, the Q^2^ values for PC, POS, and JP were 0.096, 0.052, and 0.006, respectively, all exceeding zero. Notably, although the Q^2^ value for JP exceeds zero and thus indicates nonzero predictive relevance, its magnitude is relatively small. This is consistent with the modest R^2^ observed for JP and may reflect the fact that JP is a distal outcome shaped by numerous factors beyond those captured in the present model, such as organizational- or task-level characteristics not examined in this study.

### Model fit test

According to Henseler and Sarstedt (2013), the standardized root mean square residual (SRMR)—the difference between observed and predicted correlations—serves as an absolute goodness-of-fit index, particularly suitable for analyses based on partial least squares structural equation models (PLS-SEM). Under conservative estimates, an SRMR value of less than 0.10 to 0.08 indicates a good model fit (Hair et al., 2019). The SRMR value for this model is 0.073, thereby satisfying the criterion for a good fit.

### Direct, indirect, and moderation effects

This study employed the bootstrapping method with 5,000 resampling iterations to evaluate the path coefficients and their significance within the structural model. The results indicate that JS was significantly and negatively associated with PC (*β* = −0.241, t = 4.630, *p* < 0.001) and was also negatively associated with POS (*β* = −0.321, t = 6.876, *p* < 0.001), thereby supporting the corresponding antecedent hypotheses. Additionally, PC was significantly and positively associated with JP (*β* = 0.144, t = 2.043, *p* = 0.041), while POS was similarly positively associated with JP (*β* = 0.235, t = 2.951, *p* = 0.003).

As shown in Table 7, the direct effect of JS on JP was not statistically significant (*β* = −0.064, t = 1.033, *p* = 0.302, 95% CI [−0.189, 0.058]); therefore, H1 was not supported. The nonsignificant direct effect suggests that the correlate with of JS on JP may primarily operate through the mediating mechanisms examined in this study. The indirect effect of JS on JP through POS was significant (*β* = −0.075, 95% CI [−0.133, −0.026]), supporting H2b. The indirect effect through PC was comparatively weaker (*β* = −0.035). Although the associated bootstrap *p* value was marginal (*p* = 0.073), the 95% bootstrap confidence interval did not include zero (95% CI [−0.077, −0.001]), indicating evidence of an indirect effect. Accordingly, H2a was retained for interpretation; however, this finding should be interpreted with appropriate caution because of its marginal statistical significance.

**Table 7:** Results of direct, indirect, and moderation effects.

Path	β	T	*p*	95% Bootstrap CI	Interpretation
Direct effect					
JS→ JP	−0.064	1.033	0.302	[−0.189, 0.058]	Supported
JS → PC	−0.241	4.630	<0.001	[−0.342, 0.137]	
JS → POS	−0.321	6.876	<0.001	[−0.412, 0.227]	
PC → JP	0.144	2.043	0.041	[0.003, 0.278]	
POS → JP	0.235	2.951	0.003	[0.081, 0.390]	
Indirect effects					
JS → PC → JP	−0.035	1.792	0.073	[−0.077, −0.001]	Significant indirect effect
JS → POS → JP	−0.075	2.798	0.005	[−0.133, −0.026]	Significant indirect effect
Moderation effects					
PMH × PC → JP	−0.223	3.558	<0.001	[−0.323, −0.076]	Significant Moderation
PMH × POS → JP	0.129	2.218	0.027	[0.001, 0.232]	Significant Moderation

β, standardized path coefficient. Bootstrap estimates were obtained using 5,000 resamples. Confidence intervals (95%) were used to evaluate the significance of indirect effects.

**Figure 2:**
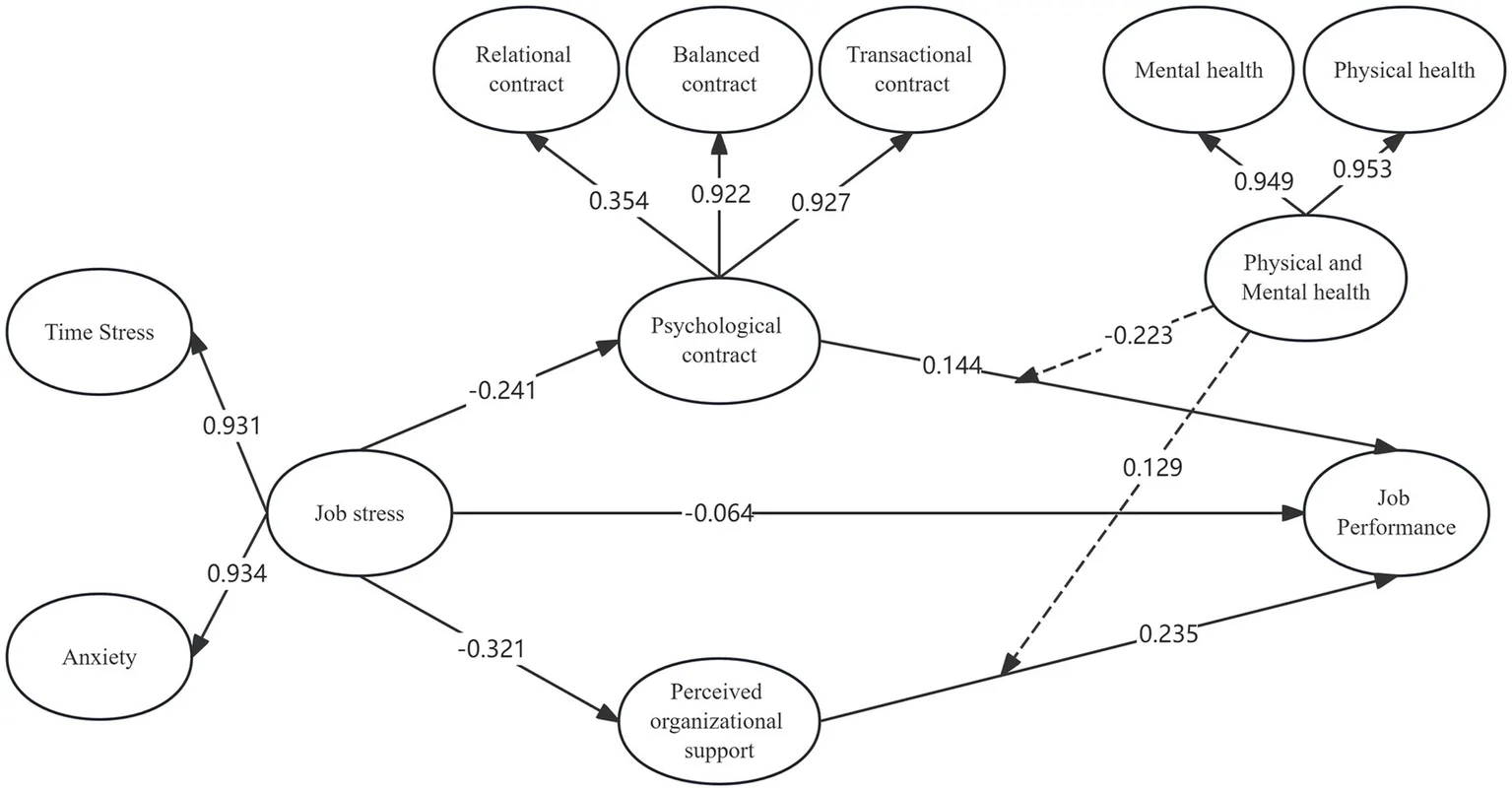
Structural equation model of the effect of job stress on job performance.

The moderating role of PMH was further examined by testing the interaction effects on the second-stage paths of the mediation model. As shown in Table 8, the interaction between PMH and PC significantly predicted JP (*β* = −0.223, t = 3.558, *p* < 0.001, 95% CI [−0.323, −0.076]), indicating that PMH significantly moderated the relationship between PC and JP. Likewise, the interaction between PMH and POS significantly predicted JP (*β* = 0.129, t = 2.218, *p* = 0.027, 95% CI [0.001, 0.232]), indicating that PMH also significantly moderated the relationship between POS and JP. Together, these findings support H3, confirming that PMH moderates both second-stage mediation paths. The opposite signs of the interaction coefficients further suggest that PMH be associated with differential moderating effects on the PC → JP and POS → JP relationships, a pattern that is discussed in greater detail in the Discussion section (Figure 2).

**Table 8:** Conditional effects across levels of physical and mental health.

Inspection content	Adjustment variable level	β	T	*p*	2.50%	97.50%	Interpretation
PC → JP	PMH (+1SD)	−0.079	0.868	0.385	−0.245	0.113	Not significant
	PMH(Mean)	0.144	2.043	0.041	0.003	0.278	significant
	PMH (-1SD)	0.367	3.750	<0.001	0.146	0.529	significant
POS → JP	PMH (+1SD)	0.364	4.494	<0.001	0.200	0.515	significant
	PMH(Mean)	0.235	2.951	0.003	0.081	0.390	significant
	PMH (-1SD)	0.106	0.930	0.352	−0.101	0.349	Not significant
JS → PC → JP	PMH (+1SD)	0.019	0.848	0.397	−0.029	0.062	Not significant
	PMH(Mean)	−0.035	1.792	0.073	−0.077	−0.001	Significant^1^
	PMH (-1SD)	−0.088	2.922	0.003	−0.149	−0.032	Supported
JS → POS → JP	PMH (+1SD)	−0.117	4.072	<0.001	−0.176	−0.064	Supported
	PMH(Mean)	−0.075	2.798	0.005	−0.133	−0.026	Supported
	PMH (-1SD)	−0.034	0.910	0.363	−0.114	0.033	Not significant

Calculated using the bootstrap resampling procedure (5,000 iterations).

### Moderation and moderated mediation effects

#### Simple slope analysis (conditional direct effects)

To further examine the second-stage moderating role of PMH, this study assessed the conditional direct effects at high (+1 SD), average (Mean), and low (−1 SD) levels of PMH. The results showed that the PC → JP and POS → JP paths exhibited opposite patterns across PMH levels (As shown in Table 8).

For the PC → JP path, the conditional direct effect was strongest and significant when PMH was low (−1 SD; *β* = 0.367, *p* < 0.001), weaker but still significant at the average level (Mean; *β* = 0.144, *p* = 0.041), and no longer significant when PMH was high (+1 SD; *β* = −0.079, *p* = 0.385).

For the POS → JP path, the pattern was reversed: the conditional direct effect was strongest and significant when PMH was high (+1 SD; *β* = 0.364, *p* < 0.001), significant at the average level (Mean; *β* = 0.235, *p* = 0.003), and no longer significant when PMH was low (−1 SD; *β* = 0.106, *p* = 0.352).

#### Test of moderated mediation (conditional indirect effects)

Building on these conditional direct effects, this study further examined the conditional indirect effects of JS on JP via PC and POS across levels of PMH (As shown in Table 8).

For the indirect path JS → PC → JP, the effect was significant at low PMH (−1 SD; *β* = −0.088, *p* = 0.003) and, based on the bootstrap confidence interval criterion, at the average level (Mean; *β* = −0.035, 95% CI [−0.077, −0.001]), but was not significant at high PMH (+1 SD; *β* = 0.019, *p* = 0.397).

For the indirect path JS → POS → JP, the pattern was reversed: the effect was significant at high PMH (+1 SD; β = −0.117, p < 0.001) and at the average level (Mean; *β* = −0.075, *p* = 0.005), but was not significant at low PMH (−1 SD; *β* = −0.034, *p* = 0.363).

These results indicate that PMH significantly moderates the second-stage paths of both mediation mechanisms, with the PC and POS pathways showing divergent patterns across levels of PMH. This divergent pattern is examined in greater theoretical depth in the Discussion section.

## Discussion

One of the key findings of this study is the divergent moderating role of PMH in the relationship between JS, parallel mediation mechanisms of PC and POS, and JP among interns. The results indicate that JS does not directly predict JP; rather, it operates through a parallel mediation path of “JS → PC/POS → JP” via PC and POS. This finding aligns with previous research indicating a negative relationship between JS and JP; however, the present results suggest that this relationship is not direct, but is instead conveyed through individual and organizational psychological perceptions as mediating mechanisms.

The nonsignificant direct effect of JS on JP (H1) necessitates careful interpretation and should not be construed as definitive evidence of full mediation alone. Several alternative explanations warrant consideration. First, this pattern aligns with cognitive appraisal theories of stress, which propose that stress rarely translates into behavioral outcomes without first being filtered through an individual’s subjective evaluation of the situation (Lazarus and Folkman, 1984; Lazurus, 1984). In this context, the absence of a direct effect may reflect the theoretical proposition that relational and health-related cognitions, rather than stress exposure per se, are the more proximal determinants of performance (Hagger, 2025). Second, an alternative statistical explanation may involve an indirect-effect-dominant pattern, wherein JS is negatively associated with both PC and POS, which are in turn positively associated with JP. Consequently, the indirect paths may partially offset a small residual direct effect, thereby attenuating its statistical significance despite a plausible underlying relationship. Third, the internship context itself may partially account for this finding; interns are typically subjected to closer supervision, structured task assignments, and less autonomy than established employees. As a result, the behavioral expression of stress may be more tightly constrained by situational factors, thereby reducing the extent to which stress directly manifests in performance variation.

This study indirectly confirms that breaches of the PC among the new generation of employees can induce burnout, and persistent negative psychological states can deplete employees’ emotional resources, hinder their focus on work, and ultimately be associated with a decline in performance (Yu et al., 2022). Furthermore, consistent with prior research, a significant positive correlation exists between POS and JP. Employees’ perceptions of POS correlate with JP (Wang, 2022), and POS is associated with enhanced JP both directly and indirectly (Uçar and Kerse, 2022). Notably, this study found that PMH exerted contrasting moderating effects across the two mediation pathways. From the perspective of Conservation of Resources (COR) theory (Hobfoll et al., 2018; Hobfoll, 1989, 2001), this pattern may reflect differences in resource investment and conservation strategies. Individuals with greater health-related resources may be more adept at investing personal resources and utilizing organizational support to manage JS, whereas those with poorer health may prioritize conserving their remaining resources to minimize further losses. It is important to note that the indirect effect of JS on JP via PC, while supported by the bootstrap confidence interval criterion, was associated with a marginal *p*-value (*p* = 0.073). This suggests that this pathway, although theoretically significant, should be interpreted with greater caution compared to the more robust indirect effect observed via POS (*p* = 0.005).

When university interns experience low levels of PMH, the positive effect of PC on JP becomes significantly more pronounced. From a situational perspective, the sample in this study—university interns—represents individuals in an early career transition stage who have not yet fully entered the workforce and are therefore undergoing career exploration and role transformation. This group generally lacks stable workplace capital accumulation. In this context, PMH emerges as an important personal resource during this transitional stage. According to resource conservation theory, when an individual’s PMH is compromised, their internal energy and emotional resources become substantially depleted (Singh and Bhuvaneswari, 2023). During such times, actively seeking, communicating, and utilizing external support from the organization (such as consulting supervisors or collaborating with colleagues) may require additional interpersonal and cognitive resources. This may create further resource depletion for individuals who are already experiencing resource loss. In contrast, PC may require relatively fewer immediate interpersonal resources for activation and retrieval. This characteristic reduces cognitive demands and provides greater accessibility under stressful conditions. Consequently, when facing low PMH and resource scarcity, individuals may increasingly rely on internalized expectations regarding the employee–organization relationship to cope with immediate JS. PC thus serves as a core relational resource for sustaining JP under conditions of resource scarcity. Furthermore, the enduring emphasis on “endurance” and the traditional virtues of “hard work and perseverance” within Chinese workplace culture may further encourage interns to interpret their current difficulties as temporary challenges within an ongoing reciprocal relationship with the organization. This interpretation may further strengthen the role of PC in sustaining individual behavior during challenging situations.

In stark contrast to the aforementioned findings, when interns exhibit higher levels of PMH, the perceived positive be associated with of organizational support on JP is significantly enhanced, while the importance of the PC is relatively diminished. This outcome aligns with the Resource Gain Spiral and Resource Complementarity effects proposed by the COR theory. Good PMH constitutes a vital personal resource. Interns with elevated levels of PMH typically demonstrate greater energy, a more positive emotional state, and enhanced adaptability to their environment (Innab et al., 2025). This heightened state enables them to be more acutely aware of the diverse support resources offered by the organization, such as mentorship, peer assistance, emotional support, and career development opportunities. More importantly, they are capable of effectively translating these external resources into tangible work behaviors and outcomes. Consequently, under conditions of robust PMH, organizational support transcends being merely an external resource; it serves as a significant catalyst for enhanced JP (Matusik et al., 2022). Conversely, when individuals experience lower levels of PMH, even if they acknowledge the presence of organizational support, they may struggle to convert it into actual performance due to a deficiency in necessary psychological energy and behavioral resources (Kim et al., 2023). In such instances, the positive effects of organizational support are challenging to fully realize, and the process of resource transformation is considerably constrained. This finding indicates that organizational support does not universally enhance JP; its effectiveness is contingent upon individuals possessing sufficient foundational health resources. In other words, PMH is a critical prerequisite for the efficacy of organizational support. This finding further delineates the boundaries of organizational support—external support is not a panacea, and its effectiveness is significantly influenced by the underlying health capital of employees.

This study demonstrates that PMH serves not only as an independent personal resource but also as a crucial foundation for determining resource allocation and transformation. Previous research has predominantly conceptualized PC fulfillment and POS as beneficial relational resources associated with positive employee outcomes (Bahadır et al., 2024). However, this study finds that the effects of these two types of resources are not fixed; they are significantly moderated by an individual’s level of PMH. Specifically, for interns with higher levels of PMH, external organizational support translates more effectively into JP; in contrast, for those with lower levels of PMH, PC provides essential psychological support for maintaining JP. This finding empirically reveals that the effects of resource substitution and resource complementarity can coexist within the same theoretical model, with their effectiveness depending on the individual’s level of basic resources, providing new empirical evidence for the application of resource conservation theory in occupational health and internship management.

These findings also help clarify the present study’s relationship to established stress-performance frameworks. Whereas the challenge-hindrance stressor framework and the Job Demands-Resources model primarily locate the buffering mechanism in job-level resources or control (Meng et al., 2023; Demerouti et al., 2001; Cavanaugh et al., 2000), the present findings suggest that individual health resources operate as a higher-order boundary condition that be a significant predictor of which of two relational-cognitive pathways—PC or organizational support—becomes functionally dominant. This extends prior frameworks by demonstrating that the mechanism through which stress be associated with performance is not fixed, but is itself contingent on individuals’ internal resource reserves, offering a more nuanced, person-centered account of the stress-performance relationship among a population, university interns, that has been comparatively underexamined in this literature.

Furthermore, this study enhances the understanding of the relationship between JS and JP. It was found that JS does not be associated with a significant direct be associated with on JP; rather, it operates through a parallel mediating path involving PC and POS. This suggests that stress does not directly diminish interns’ JP; instead, it is associated with performance through shaping their perceptions of organizational relationships and the supportive environment. Unlike previous research models, this study conceptualizes PC and POS as internal psychological resources and external organizational resources, respectively, and investigates their mechanisms of action from the perspective of a parallel resource framework. The findings indicate that college interns develop varying resource allocation preferences based on their PMH in response to stress, thereby providing a novel theoretical perspective for understanding the stress coping mechanisms of young professionals.

### Research limitations

This study investigates the moderating role of PMH, as well as the parallel mediating mechanisms of PC and POS, within the context of college interns. While the findings provide both theoretical and practical insights, several limitations should be acknowledged for future research.

Firstly, this study employed cross-sectional survey data, which, despite the statistical validation of the hypothesized path through PLS-SEM, necessitates caution in inferring causal relationships. As a result, the causal sequence among the variables remains ambiguous. The be associated with of JS on JP is a dynamic process that evolves over time. Cross-sectional data can only capture correlations between variables at a specific static point and cannot clarify this dynamic process. It is important to acknowledge that the possibility of reverse causality cannot be entirely dismissed. For instance, interns who exhibit lower performance may retrospectively report experiencing higher levels of stress or perceiving lower organizational support. Additionally, unmeasured third variables, such as personality traits or job types, may partially explain the observed associations among the study variables. Therefore, future research employing longitudinal or experimental designs is essential to rigorously establish the causal ordering of these relationships.

Secondly, the data predominantly relied on self-reported measures from college interns. Although statistical analyses indicate that the multicollinearity issue is within acceptable limits, the potential for common method bias (CMB) cannot be completely ruled out. Future research should enhance data objectivity by incorporating multi-source data, such as supervisor evaluations, mentor assessments, peer reviews, or objective performance indicators.

Third, the sample in this study focused on an internship group from a specific region, which may limit the generalizability of the results due to potential influences from cultural background and education systems. Future research should seek to cross-validate this “moderated parallel dual-mediation model” across various industries or in cross-cultural contexts to further assess the external validity and universality of the findings.

Additionally, this study examined only two resource mechanisms: PC and POS. While these mechanisms represent internal psychological resources and external organizational resources, respectively, other significant variables may have been overlooked. For instance, factors such as psychological resilience, work engagement, and emotional exhaustion could play mediating or moderating roles in the resource transformation process. Future investigations could broaden the variable structure within a more intricate resource system framework to provide a more comprehensive understanding of the stress-performance relationship.

Lastly, while this study identified the divergent moderating roles of PMH, it did not differentiate between the various dimensions of these health types (e.g., the relative contributions of mental versus physical health). Future research could refine the conceptualization of PMH to explore the distinct roles of different health dimensions in the mechanisms of resource substitution and complementarity.

## Conclusion

This study enhances the understanding of the moderating roles that PMH play in the relationships between JS, PC, POS, and JP among university interns. The results indicate that, within the internship context, JS was not directly associated with JP; rather, it was associated with JP indirectly through the PC and POS. Furthermore, were associated with significant differences in these relationships: when PMH levels are low, the link between the PC and JP becomes more pronounced; conversely, when PMH levels are high, the positive relationship between POS and JP is more prominent. These findings suggest that university interns do not uniformly utilize internal and external resources when confronted with JS; instead, they demonstrate differentiated patterns of resource dependence based on their individual PMH conditions. Individuals with lower levels of PMH tend to rely more heavily on internal psychological resources to sustain basic JP. In contrast, individuals with higher levels of PMH find that external organizational support translates more effectively into actual JP. When analyzing the JP of university interns, it is essential to consider both individual PMH and the dynamic response mechanisms to different types of resources.

## Data Availability

The original contributions presented in the study are included in the article/supplementary material, further inquiries can be directed to the corresponding author.
